# Portable Paper‐Based Nucleic Acid Enrichment for Field Testing

**DOI:** 10.1002/advs.202205217

**Published:** 2023-02-16

**Authors:** Junyang Mei, Dandan Wang, Yiheng Zhang, Dan Wu, Jinhui Cui, Mingzhe Gan, Peifeng Liu

**Affiliations:** ^1^ State Key Laboratory of Oncogenes and Related Genes Shanghai Cancer Institute Renji Hospital School of Medicine Shanghai Jiao Tong University Shanghai 200032 China; ^2^ Central Laboratory Renji Hospital School of Medicine Shanghai Jiao Tong University Shanghai 200127 China; ^3^ CAS Key Laboratory of Nano‐Bio Interface Suzhou Institute of Nano‐Tech and Nano‐Bionics Chinese Academy of Sciences Suzhou 215123 China

**Keywords:** carcinogenic infection, field‐deployable, minimum instrument requirement, paper‐based nucleic acid enrichment, point‐of‐care testing, SARS‐CoV‐2

## Abstract

Point‐of‐care testing (POCT) can be the method of choice for detecting infectious pathogens; these pathogens are responsible for not only infectious diseases such as COVID‐19, but also for certain types of cancers. For example, infections by human papillomavirus (HPV) or *Helicobacter pylori (H. pylori)* are the main cause of cervical and stomach cancers, respectively. COVID‐19 and many cancers are treatable with early diagnoses using POCT. A variety of nucleic acid testing have been developed for use in resource‐limited environments. However, questions like unintegrated nucleic acid extraction, open detection systems increase the risk of cross‐contamination, and dependence on expensive equipment and alternating current (AC) power supply, significantly limit the application of POCT, especially for on‐site testing. In this paper, a simple portable platform is reported capable of rapid sample‐to‐answer testing within 30 min based on recombinase polymerase amplification (RPA) at a lower temperature, to detect SARS‐CoV‐2 virus and *H. pylori* bacteria with a limit of detection as low as 4 × 10^2^ copies mL^−1^. The platform used a battery‐powered portable reader for on‐chip one‐pot amplification and fluorescence detection, and can test for multiple (up to four) infectious pathogens simultaneously. This platform can provide an alternative method for fast and reliable on‐site diagnostic testing.

## Graphical abstract

A simple portable platform is reported for rapid nucleic acid field testing of SARS‐CoV‐2 and *H. pylori* with minimum instruments. Including paper‐based sample processing, isothermal amplification and readout modules, it realizes the sample‐to‐answer detection within 30 min and output visual results, with the detection limit of 400 copies mL^−1^. The platform is flexible and can detect multiple pathogens in one single test.

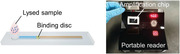

## 1 Introduction

The pandemic of infectious diseases presents an overwhelming challenge to global healthcare system. For example, the SARS‐CoV‐2 has spread all over the world and has affected more than 641 million people and resulted in more than 6.6 million deaths worldwide.^[^ ^1^ ^]^ Fast and accurate diagnostic testing with a fast turnover time is the key to effectively preventing the spread of the viruses.^[^ ^2^, ^3^, ^4^ ^]^ In addition, carcinogenic infection is an important, potentially preventable carcinogenic factor; such as *H. pylori*, HPV, and etc., are the main cause of stomach and cervical cancers, respectively.^[^ ^5^ ^]^ The infection‐induced cancers account for more than 1/6 of all cancer cases worldwide, disproportionally impact people in low‐resource settings.^[^ ^6^, ^7^ ^]^ Expanded screening in high‐risk groups could reduce cancer incidence and deaths faster and more effectively.^[^ ^8^, ^9^ ^]^ A cost‐effectively, available test is essential for managing these infections and can achieve better clinical treatment in early stages of infection.

Although sophisticated diagnostic instruments in centralized laboratories can provide highly sensitive, selective, and reproducible testing, they are often only available in hospitals of major cities and not accessible in many developing countries that lack the necessary infrastructure.^[^ ^10^, ^11^ ^]^ POCT is a simplified version of laboratory testing that has developed rapidly in recent years as a testing trend. This technology is designed to perform diagnostic analysis at or near the patient's care site, eliminating the need for complex laboratory specimen processing and quickly obtaining test results.^[^ ^8^ ^]^ Thus, they can be delivered to a variety of primary health service facilities and low‐resource areas for extended coverage of populations.^[^ ^12^, ^13^ ^]^ Nucleic acid (NA) testing, which can detect nucleic acids of microorganisms with high sensitivity and specificity, plays an important role in diagnosing various pathogens.^[^ ^14^, ^15^, ^16^ ^]^ Conventional polymerase chain reaction (PCR) requires complex temperature control, which is not suitable for on‐site testing. Isothermal nucleic acid amplifications, such as RPA and loop‐mediated isothermal amplification (LAMP), are powerful methods for POCT molecular diagnosis because they require only a constant temperature heater, like water bath or heat block, instead of a thermal cycler.^[^ ^3^, ^17^ ^]^ The RPA reaction is close to normal temperature (37–42 °C), fast and efficient, and usually only takes 20 min to produce results that can be distinguished by the naked eye.^[^ ^18^, ^19^ ^]^ It has the potential to realize portable, highly sensitive, multiplex detection in POCT development.

A variety of nucleic acid testings (NAT) have been developed for use in resource‐limited environments. For example, Jonathan Cooper group developed a paper‐based POCT device for pathogen testing through reverse transcriptase LAMP (RT‐LAMP) within 40 min.^[^ ^20^ ^]^ Lateral flow paper strip provided simple result read‐out by naked‐eyes or smartphone interpreting with deep learning algorithms.^[^ ^21^ ^]^ However, questions like unintegrated nucleic acid extraction, open detection systems increase the risk of cross‐contamination, dependence on expensive equipment (i.e., pipettes, commercial heater, centrifuge, LED transilluminator, etc.), and infrastructure (i.e., AC power supply), significantly limit the application of POCT, especially for on‐site testing.

In this paper, we described a simple portable platform for rapid infectious pathogen testing including SARS‐CoV‐2 and *H. pylori*. The samples are lysed at room temperature, and the nucleic acid is extracted using a paper‐based enrichment chip. A battery‐powered all‐in‐one portable reader is designed for both on‐chip amplification and result readout. The battery capacity can support around 25 entire batches after a single charge. To this end, we successfully detected SARS‐CoV‐2 in contrived swab samples with a limit of detection (LoD) down to 4 × 10^2^ copies mL^−1^, and *H. pylori* from contrived saliva samples with a LoD of 10^3^ counts mL^−1^. The sample‐to‐result turnaround time was shorter than 30 min in typical on‐site test settings. The results could be read with naked eyes or analyzed via a smartphone application. The platform is flexible and can detect multiple pathogens in one single test. This platform has provided a fast, accurate, and convenient way for infectious agent testing anywhere.

## 2 Results

### 2.1 Design and Operation of Our POCT Platform

**Figure 1:**
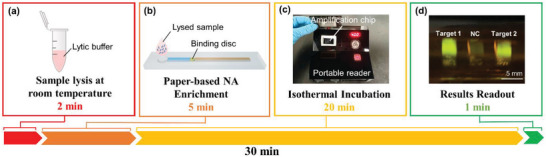
The workflow of our POCT platform. a) Lyse saliva or nasopharyngeal swab samples at room temperature. b) Paper‐based nucleic acid enrichment based on lateral flow. c) RPA reaction for 20 min at 40 °C. d) Read the result.

There are four major steps in the workflow of our sample‐to‐answer POCT (**Figure** **1**; Figure S1, Supporting Information). (1) Sample collection and lysis at room temperature. (2) Paper‐based nucleic acid enrichment. Lysed sample was loaded into the paper‐based enrichment chip, and nucleic acids were extracted to the binding disc (which was made of the GE Whatman flinders technology associates (FTA) card) through lateral flow. Then the binding disc was washed with anhydrous ethanol to quickly remove residual water. (3) RPA reaction. The binding disc, and primers, probes and RPA reagents in the preinstalled tube were loaded onto the amplification chip for one‐pot reaction. The chip was sealed with clay and incubated in the battery‐powered portable reader at 40 °C for 20 min. (4) Result readout. The chip was removed and placed in the detecting chamber and observed under 488 nm blue light excitation. The positive control showed bright green fluorescence, while the negative control remained colorless (or very light green fluorescence). The fluorescence results could be directly visualized from the portable reader and further analyzed by a smartphone application (Figure S2, Supporting Information). And see Supporting Information part 1 for more operational details.

### 2.2 Paper‐Based Sample Pretreatment

FTA cards are commonly used to extract nucleic acids for the purpose of simple, fast, and low‐cost sample preprocessing in POCT scenarios. This NA‐trapped paper can be used as an amplification substrate after drying, omitting the quantitative loading step. In general, nucleic acid containing solution is first spotted and dried up on the FTA card, and then a small piece of paper is punched off from the FTA card for subsequent nucleic acids amplification, after washing up with FTA purification reagent and TE buffer. However, only a part of the extracted nucleic acids were used for amplification in this method, requiring a relatively high concentration in the original samples for an effective pathogen detection. In order to detect low concentration of nucleic acids, we have developed a paper‐based enrichment method for sensitive, cost‐effective and easy‐handled detection.

**Figure 2:**
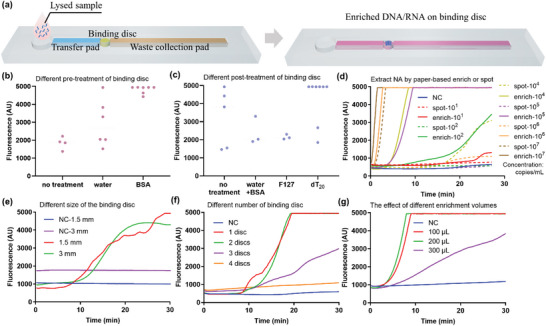
Paper‐based nucleic acid enrichment. a) Schematic of rapid paper‐based nucleic acid enrichment. b) Amplification efficiency for RNA enrichment from contrived virus samples under different pre‐treatment (DEPC water, BSA, or not) of FTA card. c) Amplification efficiency for RNA enrichment from contrived virus samples under different post‐treatment (DEPC water, BSA, F127 and oligo(dT)_20_ or not) of FTA card. d) Comparison of the amplification performance under different sample concentrations upon different nucleic acid extraction treatment. e) Binding discs of different sizes. f) Numbers of binding discs adopted on chip for enrichment. g) Sample volumes enriched on chip. (NC: Negative control. The fluorescent intensity limitation of the T8‐ISO Instrument is 5000 AU.)

A novel paper‐based nucleic acids enrichment chip was designed for fast DNA/RNA extraction. The core of the on‐chip enrichment was a 1.5 mm diameter size paper binding disc (punched from FTA cards), bridging the transfer and waste collection pads (**Figure** **2**a). A volume of 100 µL of sample cleaved using lystic buffer at room temperature was added to the sample loading area and drawn up the chip via capillary action across the binding disc. The nucleic acids were captured on the binding disc, and the waste was collected in the waste pad. We dried the binding disc with the captured nucleic acid and used it directly as an amplification substrate for the RPA reaction. Within 30 min, fluorescence amplification signal was detected from samples with the nucleic acid concentration as low as 10^2^ copies mL^−1^ using our paper‐based enrichment method, whereas the lower limit of sample concentration for the common FTA card extraction method was 10^4^ copies mL^−1^. Our paper‐based enrichment method was 10^2^ times more sensitive than the common FTA card extraction method (Figure 2d). A possible explanation is that more target nucleic acids in the sample was trapped in the binding disc while the amplification inhibitors were washed off through lateral flow.

We designed and manufactured three enrichment chips using glass, polydimethylsiloxane (PDMS) and polymethyl methacrylate (PMMA) as support materials respectively (Figure S3a, Supporting Information). The binding disc was placed in the middle, and the transfer pad and the waste collection pad made of regular household tissue paper were attached on each side of the binding disc respectively, all through physical contact. The surface of the glass material was prone to a short liquid circuit, so the enrichment performance was unstable (Figure S3b, Supporting Information). In contrast, the sample liquid was confined in the paper flow channel and can only flow through the binding disc due to the hydrophobic PDMS wall and the hollow bridge of the PMMA chip, and the enrichment effect of these two chips was excellent (Figure S3b, Supporting Information). However, the flow rate was slow in the PMMA chip because of the hollow bridge structure in the middle of the PMMA chip. The PDMS chip had a fast liquid flow rate with no short flow, and it was selected for our subsequent experiments. Furthermore, due to the flexibility of PDMS, when the disc and paper are put in grooves on a chip, slight deformation at the brim of disc and paper occurs. It is easy for walls of grooves to catch disc and paper by friction caused by the contact of walls and disc and paper.

FTA cards are generally used to preserve DNA while their performance in the detection of RNA viruses was not ideal. We found that the extraction efficiency of paper‐based enrichment for RNA from contrived swab samples was poor and unstable when the sample concentration was lower than 10^4^ copies mL^−1^ (Figure S4a,b, Supporting Information). We have tried to purify RNA inhibitors in samples with FTA purification reagent and FTA Elute card, but the results are not satisfactory (see Supporting Information for details). Finally, we passivated the binding disc pre‐ or post‐RNA extraction with different reagents such as DEPC water, 1% albumin bovine (BSA), oligo(dT)_20_, 0.2% poloxamer (F127), etc. The results showed that FTA cards prepassivated with BSA or postpassivated with oligo(dT)_20_ could stabilize the detection performance for RNA samples at 10^4^ copies mL^−1^ (Figure 2b,c). Because the pretreatment by oligo nucleic acids may weaken the RNA capture of cellulose, facilitating the RNA releasing from paper for next amplification.

Another critical issue of sample pretreatment is lysis. The conventional bacterial or viral cracking method is heating at 95 °C, which requires high energy consumption and risks scalding. Therefore, it is highly desirable for a rapid and room‐temperature lysis method without heating, combining with the paper‐based nucleic acid enrichment. We tested some common lysis buffers (like Trizol, RIPA, NP‐40, and TritonX‐100) for contrived bacterial or viral samples at room temperature. The results showed that both Trizol and RIPA performed well for contrived viral samples (Figure S6a, Supporting Information), and both RIPA and NP‐40 worked well for contrived bacterial samples (Figure S6b, Supporting Information).

We also experimentally verified that one 1.5‐mm‐diameter binding disc was sufficient for the nucleic acid enrichment. Larger or more binding discs did not significantly improve the performance (Figure 2e,f). Furthermore, we optimized the appropriate sample volume for enrichment. A volume of 100 µL of lysed samples enriched on a 1.5 mm binding disc was adequate for a satisfactory amplification in 8 min of enrichment time (Figure 2g).

### 2.3 Construction of the Portable Reader

**Figure 3:**
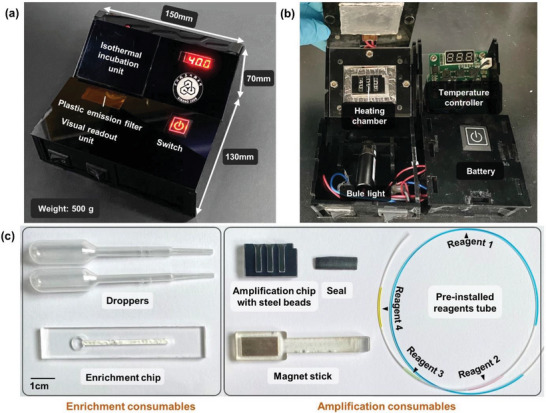
Composition of the POCT platform. a) The appearance of the portable reader. b) The interior construction of portable reader. c) Consumables used for the POCT detection.

In fact, after nucleic acid extraction, the NA‐loaded filter paper can be used for subsequent RPA reaction and result reading in many ways. For example, the T8‐ISO constant temperature fluorescence instrument can monitor the fluorescence signal in real time during isothermal incubation, which is suitable for temporary detection points or laboratories. Although simple to use, such commercial instruments are expensive (about twenty thousand dollars) and require AC power, which can limit their application in POCT scenarios. Therefore, we designed and built a rechargeable portable reader for constant temperature heating and end‐point fluorescence output (**Figure** **3**a,b). The portable reader was compact and lightweight (15 cm × 13 cm × 7 cm in size and weighs only 500 g). With dual rechargeable Li‐batteries, a single charge can keep it running for 8–9 hours and support at least 24 batches of tests. The laboratory manufacturing cost is less than $30 (Table S2, Supporting Information). And the portable reader does not need to be replaced or cleaned after each use.

An isothermal incubation unit, a visual readout unit, a temperature controller and dual 18650 batteries were integrated into the battery‐powered portable reader (Figure 3a,b). The isothermal incubation unit was mainly two heating pads, controlled by a temperature circuit, that set both at the bottom of the heating chamber and on the lid, for planar heating of the amplification chip (Figure S10, Supporting Information). The visual readout unit was a 488 nm blue light and a plastic emission filter (Figure S11, Supporting Information). The plastic filter is an emission light filter, passing the green flurorescence and blocking the exciting wavelength from the lamp.

### 2.4 On‐Chip One‐Pot Visual Detection Using the Portable Reader

We adopted one‐pot amplification on a chip, and the whole reaction was conducted in a sealed system with no need to open the lid, which can effectively avoid false positive results caused by environmental aerosol contamination.^[^ ^2^, ^22^, ^23^ ^]^ Due to the strong reflection of the circular PCR tube during fluorescence reading, high‐quality filter was needed to assist observation. Since high‐quality filters were expensive and usually cost tens of dollars or more, we tried using regular plastic emission filter as substitutes, but the separation efficiency of light was not high. For that reason, we designed a planar amplification chip (Figure 3c; Figure S13a, Supporting Information). The planar amplification chip was made of three layers of PMMA plate, and the background negative plate was a black plate to reduce light reflection and improve the signal‐to‐noise ratio. The plastic plates are low‐cost and can be mass‐produced, and the number of samples in one batch of test can be changed flexibly by altering the design of the chip.

**Figure 4:**
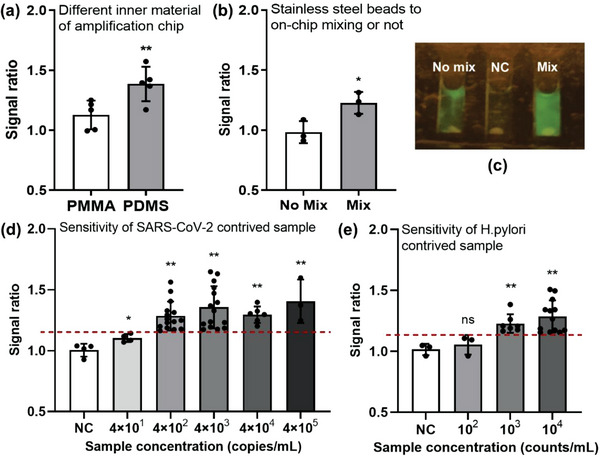
The detection performance of our POCT platform using contrived samples. a) Amplification chips made of PMMA or PDMS (*n* = 5). b) With/without mixing by stainless steel beads (*n* = 3). Pseudoviruses by packaging SARS‐CoV‐2 RNA was used for input. c) The picture of with/without mixing by stainless steel beads. The SARS‐CoV‐2 RNA standard was used for input. d) Detection sensitivity for contrived SARS‐CoV‐2 samples (*n* ≥ 3). e) Detection sensitivity for contrived *H. pylori* samples (*n* ≥ 3). (The signal ratio represents the ratio of fluorescence values of positive and negative controls in a batch of tests. The signal ratio of NC represents the ratio of the fluorescence values of each test in the total negative control batch. The value of average fluorescence intensity of negative control plus 3 × standard deviation (SD) which was used as the cut‐off value for a positive signa. Error bars represent the means ± SD of replicates. (ns: not significant, **P < 0.01, *P < 0.05, t‐test).

To complete fast RPA on chip, two major problems, strong nonspecific adsorption on chip surface and inadequate mixing, should be solved. Considering the fact that the RPA reaction could be inhibited due to the strong adsorption of proteins on the surface of untreated PMMA,^[^ ^24^ ^]^ the inner surface of the amplification chip was passivated with 2% BSA, oligo(dT)_20_ (5 × 10^−6^ m) and 0.2% F127. It was found that the passivated chip could detect contrived samples at the concentration of 10^3^ copies mL^−1^ or higher, but still no amplification of fluorescence signal was observed in samples with lower concentrations (Figure S13b, Supporting Information). Therefore, an additional layer of PDMS, a suitable material for PCR chip fabrication, was applied to the chip chambers' interior surface before passivation with BSA and oligo(dT)_20_.^[^ ^25^, ^26^ ^]^ We found that the fluorescence signal intensity after amplification for our contrived samples at 10^3^ copies mL^−1^ was significantly higher than that of amplification chips using PMMA as inner layer material (*P* < 0.01) (**Figure** **4**a).

Since the RPA system is relatively viscous, it is recommended to stir the reaction during the isothermal incubation when higher sensitivity detection is required. To this end, we added stainless 4–6 steel beads with a diameter of 0.6 mm to the reaction system. Before and after the constant temperature incubation, the steel beads were stirred twice with a magnet stick, which was moved several times from top of the chip to bottom and then back, to mix the system. As shown in Figure 4c, under the same conditions, mixing by stainless steel beads stirring can accelerate the amplification reaction and make the fluorescence intensity higher and more uniform, and the difference in fluorescence intensity was statistically significant (*P* < 0.05) (Figure 4b).

### 2.5 Performance of the Platform Using Contrived Samples

Our platform has provided a complete on‐site testing solution. To evaluate the performance of this platform, a protocol for the detection of contrived bacterial or viral samples was developed. We detected SARS‐CoV‐2 contrived nasopharyngeal swab samples at different concentrations, and the LoD was shown to be as low as 4 × 10^2^ copies mL^−1^ (Figure 4d), comparable to that of PCR method.^[^ ^4^, ^22^, ^27^ ^]^ In other words, this portable platform was sensitive enough to perform diagnostic testing for typical SARS‐CoV‐2 clinical samples.^[^ ^28^ ^]^ This POCT platform can be used to detect not only SARS‐CoV‐2 but also carcinogenic infection. We tested with contrived *H. pylori*, the most common carcinogenic microorganism, samples to evaluate the performance of this platform. And the LoD was shown to be as low as 1 × 10^3^ counts mL^−1^ (Figure 4e), similar to the sensitivity of PCR test, and capable of detecting *H. pylori* in typical clinical saliva samples.^[^ ^29^, ^30^ ^]^ In addition, the coefficient of variation (CV) values was all smaller than 15%, showing that the test results were reliable (Tables S3 and S4, Supporting Information). This portable platform was capable of performing the whole testing process with minimal equipment, and the sample‐to‐result turnaround time was shorter than 30 min, making it an ideal tool for on‐site diagnostic testing.

### 2.6 Smart Phone Assisted Reading and Sharing of Test Results

Given that visual detection occasionally suffers from operator bias, visual identification becomes difficult when sample concentrations are low (usually when the concentration close to the LoD). Therefore, we developed a smartphone‐based assisted fluorescence analysis channel. The user first takes a fluorescence image of the end point with the phone's camera. A mobile phone application developed in conjunction can calculate the ratio of the pixel intensity distribution of the positive control tube and the negative control tube, and return a result indicating whether the target pathogen exists in the sample (Figure S2, Supporting Information). People can also upload images to supporting website (see the Supporting Information for details). With user permission, the online tools can read the images' metadata, such as time and coordinates, and direct feedback to medical and health institutions, so that positive patients can receive treatment quickly and reduce privacy leakage in intermediate links (Figure S15, Supporting Information). It also can plot the geo‐distribution of positive tests and the trend over time (Figure S16, Supporting Information), allowing the users to track the spread of the specific virus.

## 3 Discussion

This paper demonstrates a simple portable platform capable of rapid sample‐to‐answer testing within 30 min to detect carcinogenic microorganisms (like *H. pylori*) or other pathogens (like SARS‐CoV‐2). The battery‐powered portable reader integrated isothermal incubation unit, visual readout unit and prestored reagents consisting of a full on‐site diagnostic testing package. This POCT platform ensured a rapid sample‐to‐answer turnaround time (less than 30 mins) with a LoD as low as 4 × 10^2^ copies mL^−1^ for SARS‐CoV‐2 and 10^3^ counts mL^−1^ for *H. pylori* in contrived samples. The all‐in‐one portable reader is small, easy to carry, and can support tests for more than 24 batches after a single charge. The results outputted by the portable reader can be either directly visualized and analyzed via a smartphone application. Test results can be instantly shared so that the spread of certain infections can be monitored in real time. Moreover, by using different primers or probes for different targets, this platform can also be adapted to perform diagnostic tests for other infectious pathogens.

In order to realize sample pretreatment in POCT scenario, we developed a paper‐based enrichment method that allows quick nucleic acid extraction from raw swabs or saliva samples. We optimized and screened a series of conditions for paper‐based enrichment, including the design and construction of enrichment chip, pre‐ and post‐processing methods for FTA cards to improve the stability of RNA extraction, and etc. Combined with sample cleavage at room temperature, our pretreatment method achieved excellent sensitivity in subsequent RPA amplification. In addition, the RIPA lysate has the same performance as Trizol and can handle bacterial or viral samples, without toxicity, which is more in line with POCT requirements.

The amplification method is a triple step amplification using coupled RPA from a paper‐based enrichment chip. In more detail, nucleic acids from the lysed sample were first extracted and enriched onto the binding FTA disc through lateral flow. This is the first amplification. Then the enriched and extracted nucleic acids were transferred to the preinstalled tube which was loaded onto the amplification chip. The second amplification was through RPA by introducing an exo FRET probe for fluorescence detection. The third amplification was completed through adding stainless‐steel beads for agitating the system twice since accumulating amplicons of the reaction may in particular benefit from agitation.

Several fully integrated isothermal nucleic acid amplification test devices have been reported. Most of them are based on the LAMP, which requires an incubation of ≈60 min at around 60 °C or higher.^[^ ^31^, ^32^, ^33^, ^34^, ^35^, ^36^ ^]^ This relatively long period of maintaining a high temperature consumes much energy, resulting usually a single usage or only a few usages after multiple recharging which effective increases the time needed, the final cost, and the wider applications especially in resource limited stings. Our three step amplifications with RPA performs optimally at 37–42 °C, consuming much less energy. With two chargeable batteries, our device can sustain a maximum 24 batches of tests before recharging.

On the other hand, extraction free sample can be adopted in some LAMP‐based testing, which make the operation easier.^[^ ^37^ ^]^ Yet, the target number loading into the amplification reaction is restricted. For the small concentration of sample, it requires more sample volume, thus may dilute the amplification reagent. Although increasing the total reaction volume could counteract over dilution, it will add reagent cost in single test. The paper enrichment could increase the target loading, captured from more sample volume, and enhance the sensitivity without dilution of the reaction system. Besides that, unlike the engineered Bst polymerase with strong inhibitor tolerance, the constitution of RPA is more complex and susceptible to inhibitors in sample, especially close to the limit of detection.^[^ ^38^ ^]^ The paper‐based enrichment offering purified DNA/RNA, provides the stability of RPA.

In addition, our system provides a much shorter turnaround time of about 30 min with comparable or better sensitivity. These advantages are in particular important and significant improvement over other reported test systems for those pathogens that require both frequent and rapid tests in community such as SARS‐CoV‐2.

Further advances will be required for our platform to execute in any location, including in at‐home or outdoor settings. First, there are several manual steps in enrichment, reagents addition and results reading. Thus, it was more fit to the trained users or amateurs by simple guidance, rather than nontrained users. Second, all the enzymes need cold storage and cold‐chain transportation. In addition, portable readers are not yet small enough to be carried around in a pocket. These limitations may hinder a wider adoption of the reported platform. In future work, we will integrate manual steps, take out the cold chain transmission, and narrow down the detection device, to expand custom and application scopes.

## 4 Conclusion

In summary, a portable platform is developed for fast and accurate on‐site diagnostic testing for infectious pathogen. Compared with other representative POCT methods (Tables S5 and S6, Supporting Information), it is a long‐endurance unplugged detection system, that can perform 25 batches of testing on a single charge. Moreover, it can quickly extract nucleic acids at room temperature, simple to use, fast and sensitive (the detection limit can reach 4 × 10^2^ copies mL^−1^ within 30 min), and has a low equipment cost (the laboratory manufacturing cost is less than $30). Multiple pathogens can be detected simultaneously (including saliva or swab samples, bacterial or viruses). The application of this platform will significantly facilitate on‐site infectious pathogen testing, especially in those under‐developed regions of the world.

## 5 Experimental Section

### Materials

Template‐wrapped lentivirus was synthesized by Fubio Biological Technology (China). Plasmid, primers, probes, and template‐encapsulated *E. coli* were synthesized by Sangon Biotech (China). oligo(dT)_20_ and synthetic RNA were synthesized by Hongxun Bio (China). RevertAid reverse transcriptase was purchased from Thermo Fisher Scientific (US). RNase inhibitor, Trizol, RIPA, TE buffer, NP‐40, TritonX‐100, PBS, dithiothreitol (DTT), and DEPC treated water were purchased from Beyotime (China). TwistAmp kit was purchased from TwistDx Limited (UK). The binding disc was made of the GE Whatman FTA card, and the FTA purification reagent was purchased from Whatman (UK). BSA was purchased from Solarbio (China). F127 was purchased from Sigma–Aldrich (China). Anhydrous ethanol was purchased from Titan (China).

### Chip Fabrication

The enrichment chip was made of PDMS with a binding disc slot and flow channels. The amplification chip (with three or more amplification chambers) was made of PMMA with a layer of PDMS coated to the interior surface of the chambers. The surface of the enrichment chip and the inside of the amplification chip were passivated with 2% BSA, 0.2% F127, and 5 × 10^−6^ m oligo(dT)_20_ for about 2 hours. Four to six steel beads with a diameter of 0.6 mm were added to each amplification chamber.

### Paper‐Based Nucleic Acid Extraction

For paper‐based DNA enrichment, according to the schematic shown in Figure 2a, the nucleic acid was enriched into the binding disc through lateral flow and then rinsed with 10 µL anhydrous ethanol to quickly volatilized the moisture. For paper‐based RNA enrichment, the extraction steps were the same as those for DNA enrichment, except that the binding discs need to be soaked in 1% BSA and thoroughly dried for reservation prior to RNA extraction. After RNA enrichment, excess NA binding sites on the binding disc were blocked by oligo(dT)_20_ (10 × 10^−6^ m).

### Contrived Samples Preparation

Lentivirus‐wrapped template was used as contrived sample for SARS‐CoV‐2. 10 µL RNase inhibitor and 10 µL DTT was add to 50 µL Trizol or RIPA, and the resulting solution was agitated gently with a NP swab following the addition of 50 µL pseudovirus. *E. coli*‐encapsulated template was used as contrived sample for *H. pylori*. 10 µL glycerobacteria was mixed with 30 µL saliva and 60 µL RIPA for lysis. Then DNA was enriched into the binding disc. Saliva samples from healthy humans were collected from all authors, approved by the ethics committee of Suzhou institute of nano‐tech and nano‐bionics (SINANO/EC/2022‐010).

### RPA Reaction

The Urease B subunit (UreB) from *H. pylori* and virus‐specific ORF1ab from SARS‐CoV‐2 were chosen as the targets for detection. Amplification primers were designed according to the above nucleic acid sequences (Table S1, Supporting Information). RNase P gene was used as internal control (IC) to avoid possible false‐negative results.^[^ ^39^ ^]^ For RPA amplification, 29.5 µL rehydration buffer, 4.8 µL primer mix (containing reverse and forward primer), 0.6 µL exo probe and 13.2 µL DEPC‐treated water were mixed to reconstitute the freeze‐dried reaction pellet. For reverse transcription‐RPA (RT‐RPA) reaction, 29.5 µL rehydration buffer, 4.2 µL primer mix, 0.6 µL exo probe, 9.45 µL DEPC‐treated water, 1.25 µL RNase inhibitor, and 2.5 µL reverse transcriptase were mixed, and 2.5 µL magnesium acetate was add in the last step to trigger the reaction.

### Result Readout and Data Analysis

Fluorescence images were photographed with smartphones and saved in JPG format. Fluorescence intensity was measured on Image J, followed by calculating the fluorescence signal ratio of tested samples to the negative control. Statistical analysis was performed using GraphPad Prism 7.0 software. The test result was defined as positive if the fluorescence intensity was more than 3× standard deviation (SD) above the average fluorescence intensity of the negative control. The end point fluorescence data were displayed with error bars representing mean ± SD from three or more replicates. The unpaired two‐tailed *t*‐test was applied to investigate the difference between groups, and the difference was considered significant when *p* value <0.05.

## Conflict of Interest

The authors declare no conflict of interest.

## Author Contributions

J.M. and D.W. contributed equally to this work. P.L., M.G., and J.C. conceived and designed the study; J.M., D.W., Y.Z., and D.W. carried out experiments; J.M., D.W., and Y.Z. wrote the manuscript. All authors commented on the manuscript.

## Supporting information

Supporting Information

## Data Availability

The data that support the findings of this study are available on request from the corresponding author. The data are not publicly available due to privacy or ethical restrictions.
