# Supplementary material for: Portable Paper‐Based Nucleic Acid Enrichment for Field Testing
Source: Adv Sci (Weinh). 2023 Feb 16;10(11):2205217. doi: 10.1002/advs.202205217 (PMC10104631; doi:10.1002/advs.202205217)
Supplement: Supplementary file 1 — Supporting Information [file ADVS-10-2205217-s001.pdf]

ADVANCED SCIENCE
Open Access

Supporting Information

for *Adv. Sci.*, DOI 10.1002/advs.202205217

Portable Paper-Based Nucleic Acid Enrichment for Field Testing

Junyang Mei, Dandan Wang, Yiheng Zhang, Dan Wu, Jinhui Cui, Mingzhe Gan* and Peifeng Liu**

Electronic Supplementary Information

Portable paper-based nucleic acid enrichment for field testing

*Junyang Mei, Dandan Wang, Yiheng Zhang, Dan Wu, Jinhui Cui *, Mingzhe Gan *, Peifeng Liu **

List of Contents

1. Operation of Our POCT Platform
2. Optimization of Paper-based Sample Pretreatment
3. Optimization of Recombinase Polymerase Amplification (RPA) Reaction
4. Construction of the Portable Reader
5. Construction of Amplification Chip
6. Performance of the Platform Using Contrived Samples
7. Smart phone assisted reading and sharing of test results
8. Comparison with some typical point-of-care detection work

1. Operation of Our POCT Platform

More detailed operation processes are shown in the Figure S1.

Sample collection: The collected swab or saliva samples are added to lytic buffers for simple lysis at room temperature (Figure 1a).

Paper-based nucleic acid enrichment: About 100 μL of the lysed sample was absorbed with an dropper and added to the sample loading area of the enrichment chip. Nucleic acids were extracted to the binding disc (which was made of the GE Whatman FTA card) through lateral flow. Then the binding disc was washed with anhydrous ethanol to quickly remove residual water. Then 20 μL of anhydrous ethanol is added to the sample loading area, allowing the binding disc to quickly dry in 2 minutes. If the target is RNA, 20 μL oligo$(dT)_{20}$ (10 μM) is added to the sampling site prior to this step to weaken the RNA capture of cellulose, facilitating the RNA releasing from paper for next amplification.

Recombinase polymerase amplification (RPA) reaction: The dried binding disc extracted nucleic acids was transferred to the amplification chamber of the amplification chip. Primers, probes and other RPA reagents in the pre-installed tube were loaded onto the amplification chamber for one-pot reaction. Pre-installed reagent tube with several amplication reagents spacing by air gap. All solutions are separated by air gap and are easily pushed out sequentially using a dropper. The chip was sealed with clay and incubated in the heating chanmber of the portable reader at 40 °C for 20 minutes. Before and after the constant temperature incubation, the steel beads were stirred twice with a magnet stick, which is moved several times from top of the chip to bottom and then back, to mix the system.

Result readout: The chip was removed and placed in the detecting chamber and observed under 488 nm blue light excitation. The fluorescence results could be directly visualized from the portable reader and further analyzed by a smartphone application (Figure S2).

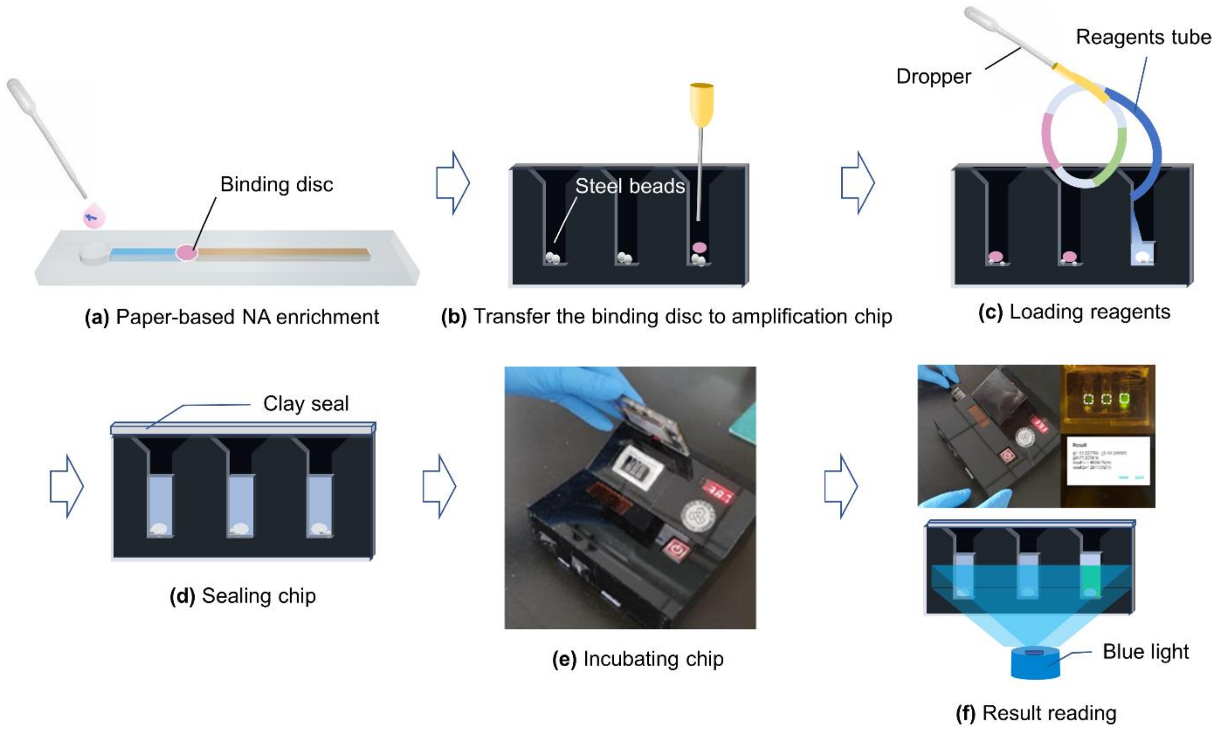

Figure S1. The operation details of our POCT platform. (a) Paper-based NA enrichment after sample lysis. (b) Transfer the binding disc from enrichment chip to amplification chip. (c) Primers, probes and RPA reagents were loaded onto the amplification chip for one-pot RPA reaction. (d) Seal the amplification chip by clay. (e) Incubate the amplification chip at 40 °C for 20 minutes. (f) Readout under bule light.

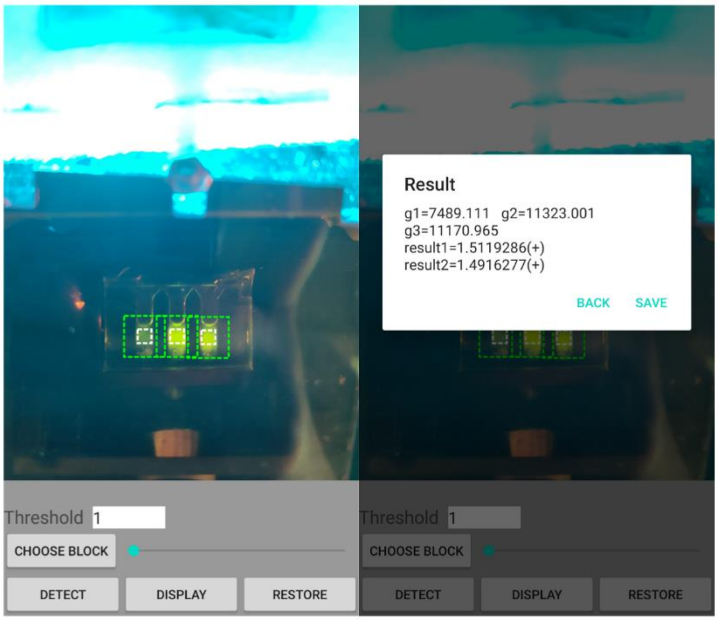

Figure S2. Quantitate the fluorescence intensity and determine the test result on the smartphone.

2. Optimization of Paper-based Sample Pretreatment

In view of the unstable effect of the paper-based nucleic acid enrichment method extract RNA from low concentration samples (< 10^4 copies/mL) (Figure S4a, b), we first considered the purification of RNA inhibitors in the sample. We tried washing the binding disc with the FTA purification reagent to reduce the influence of RNase and amplification inhibitors, but the fluorescence signal intensity did not improve (Figure S4c). Then we try to add an FTA Elute card before the FTA card in the flow channel (Figure S5). As the sample fluid flows through the FTA Elute card, the repressor proteins are tightly bound, and the RNA is captured by the FTA card downstream, to further purify RNA. However, the amplification signal intensity was not improved either (Figure S4d).

Cellulose can strongly capture the nucleic acids and retain them following purification steps [1].Typical RNA amplification requires an additional elution step to release RNA from the paper into the amplification solution [2]. Considering the omitted elution step and the small amount of target RNA in our procedure, RNA may be strongly captured inside the paper and difficult to be released into the solution. Thus, it may be insufficient to trigger the next amplification. We designed the passivation steps by BSA and oligo nucleic acids to pre-occupy the cellulose and/or to weaken the RNA binding via competition, effectively facilitating the RNA releasing from paper for the next amplification step. Through repeated experiments, we found that the FTA card was blunted by pre-soaking with 1% BSA. After RNA extraction, the remaining nucleic acid binding sites in FTA card were blocked by single stranded oligo$(dT)_{20}$ (10 μM), weaken the RNA capture of cellulose, facilitating the RNA releasing from paper for next amplification. This treatment method could stably detect 10^4 copies/mL of RNA in samples with low concentration (Figure 2b,c). And pre-passivation with higher concentration (10%) of BSA did not further increase the amplification efficiency and BSA might precipitate when contacting the lysis buffer (Figure S4e).

After nucleic acid extraction, the binding disc must be dried for further amplification. It took about 20 minutes for our binding discs to dry naturally at room temperature. Heating up the binding discs by a handwarmer can shorten this time to 8 ~ 10 minutes. We also tried rinsing the binding discs with anhydrous ethanol to quickly volatilize the moisture. The effects of different drying methods were compared (Figure S6c). Anhydrous ethanol rinsing can dry the binding disc in only 1 ~ 2 minutes with no adverse effects on subsequent nucleic acids amplification.

By optimizing the extraction conditions (see Results 2.2 for more details), our paper based nucleic acid enrichment method were able to detect SARS-CoV-2 RNA in the contrived swab

virus samples with a LoD of 400 copies/mL (Figure S7b), and *H. pylori* DNA in the contrived saliva samples with a LoD as low as 10^3 counts/mL (Figure S7a).

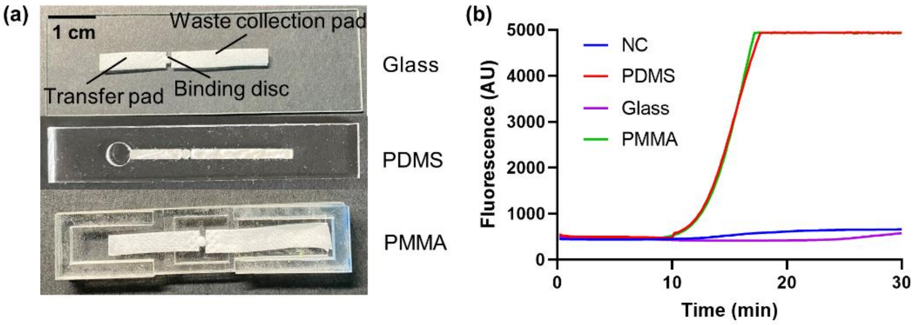

Figure S3. Design and extraction effect of enrichment chip. (a) The picture of three different enrichment chips (Glass, PDMS and PMMA). (b) The amplification efficiency of these three enrichment chips.

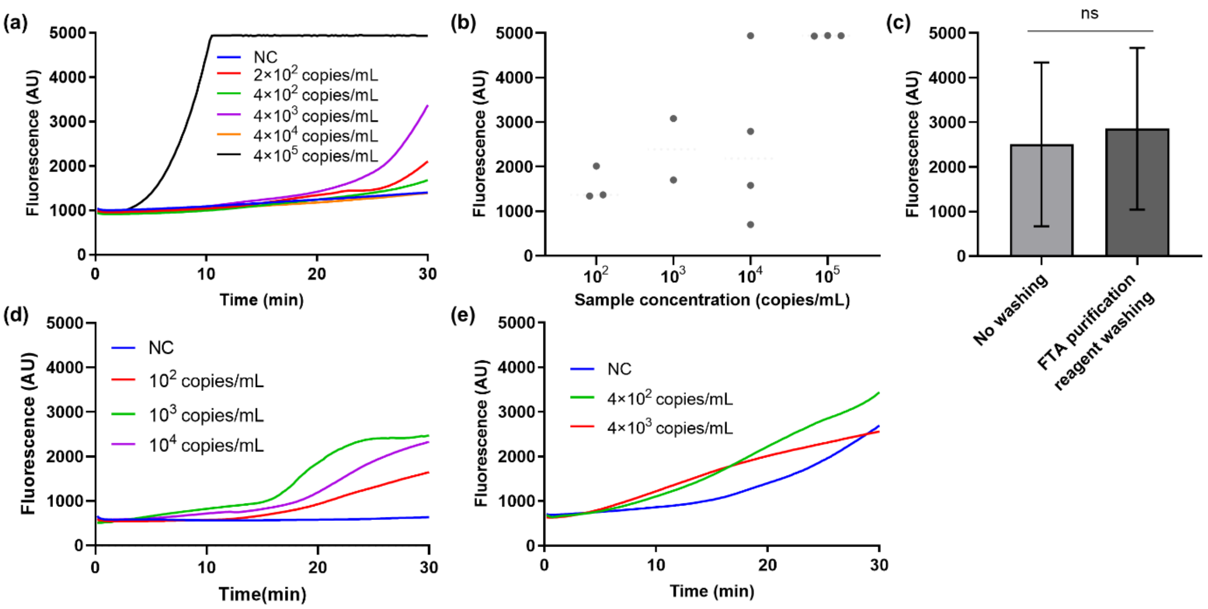

Figure S4. Optimization of paper-based RNA enrichment. (a,b) Amplification efficiency for RNA extracted from contrived SARS-CoV-2 samples under different concentrations. (c) Amplification efficiency for RNA extracted from contrived SARS-CoV-2 samples with/without FTA purification reagent washing. Three replicates were run (n=3). (d) Amplification efficiency for contrived SARS-CoV-2 samples under different concentrations when adding an FTA Elute card before the FTA card in the flow channel. (e) Amplification efficiency under pre-treatment with higher concentration (10%) of BSA. Error bars represent the means ± SD from replicates. (ns: not significant, t-test).

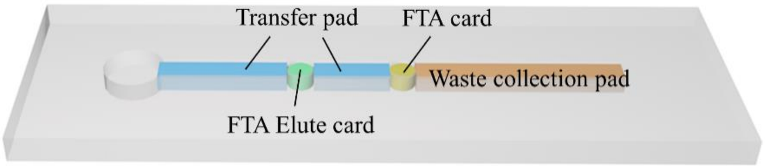

Figure S5. Add an FTA Elute card before the FTA card in the enrichment chip flow channel.

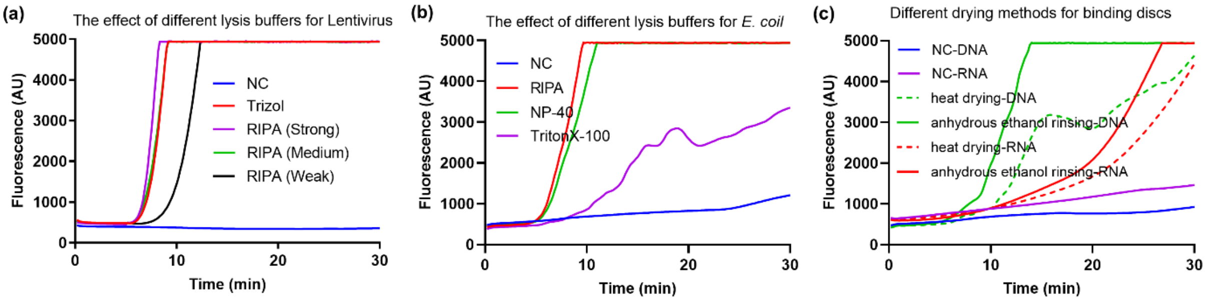

Figure S6. Optimization of the lytic buffer and drying conditions for binding disc. (a) Amplification efficiency for pseudovirus upon different lytic buffer treatment. (b) Amplification efficiency for bacteria vector upon different lysis buffer treatment. (c) Drying methods of binding discs including anhydrous ethanol rinsing and heating.

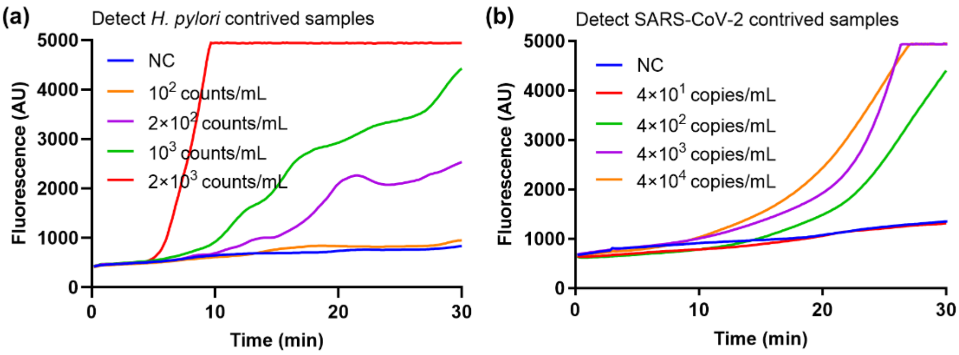

Figure S7. Performance of nucleic acid extraction by paper-based enrichment. (a) The extraction performance of contrived *H. pylori* saliva samples. (b) The extraction performance of contrived SARS-CoV-2 swab samples.

3. Optimization of Recombinase Polymerase Amplification (RPA) Reaction

To optimize the RPA reaction, we introduced exo probe for real-time detection of amplification by fluorescence. Probes intended for the use in PCR and other nucleic acid amplification processes (e.g. Taqman®) will not work in RT-RPA exo reactions. Therefore we designed and screened of a series of candidate primers and probes, probe concentrations. We evaluated their ability to produce an effective amplification of nucleic acids samples with 30 minutes of incubation through fluorescence instrument (Figure S8). After optimization of

parameters, we found that 120 nM of the probe worked well (Figure S8a). The reverse transcriptase activity is essential for RNA detection, especially at low RNA concentrations. So, we screened of the reverse transcriptase and selected RevertAid for next test because of its good performance (Figure S8b). Furthermore, detection of RNA typically requires reverse transcription and amplification that can be performed sequentially (two-step) or simultaneously in a one-pot system (one-step). Despite that the two-step reaction was more sensitive than one-step reaction as revealed by our results, the one-step method, omitting the opening of the chip, was easier to handle and time saving (Figure S9a, b).

In addition, human ribonucnase P gene (RNase P) was selected as an Internal control (IC) to monitor the quality of samples collected and the effectiveness of sample pretreatment, namely the accuracy of the experimental operation process, to avoid possible false negative results [3-5]. Multiple pairs of primer pairs were designed for *H. pylori*, SARS-CoV-2 and RNase P respectively, and the combination with high amplification efficiency was selected, as shown in Figure S8c, d and Table S1.

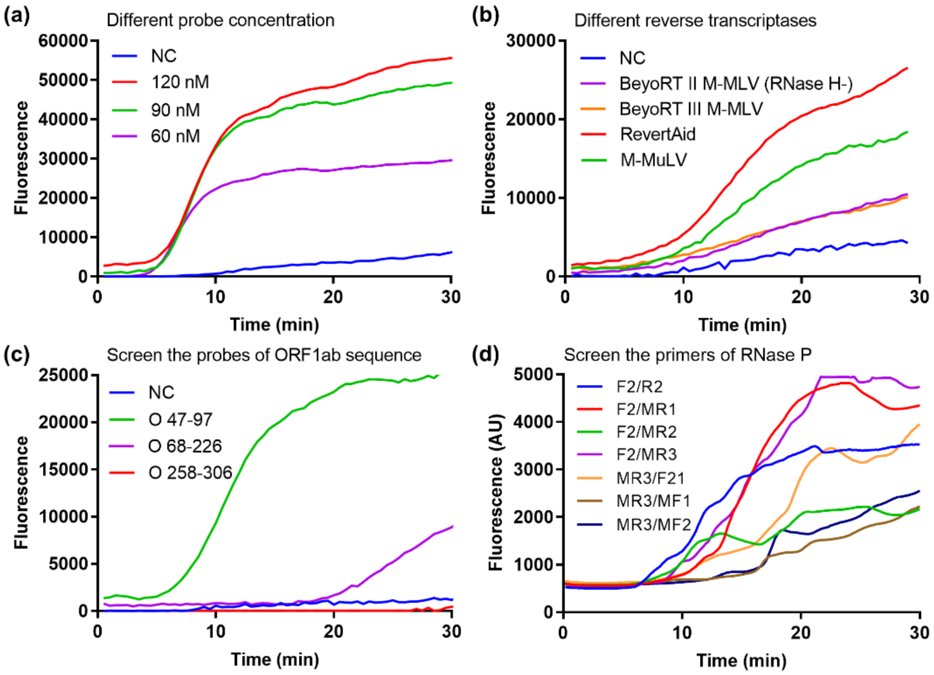

Figure S8. Construction and optimization of RT-RPA reactions. (a) Screen the concentration of probes. (b) Screen the reverse transcriptase of RT-RPA reactions. (c) Screen the probes of the ORF1ab sequence. (d) Screen the primers of RNase P.

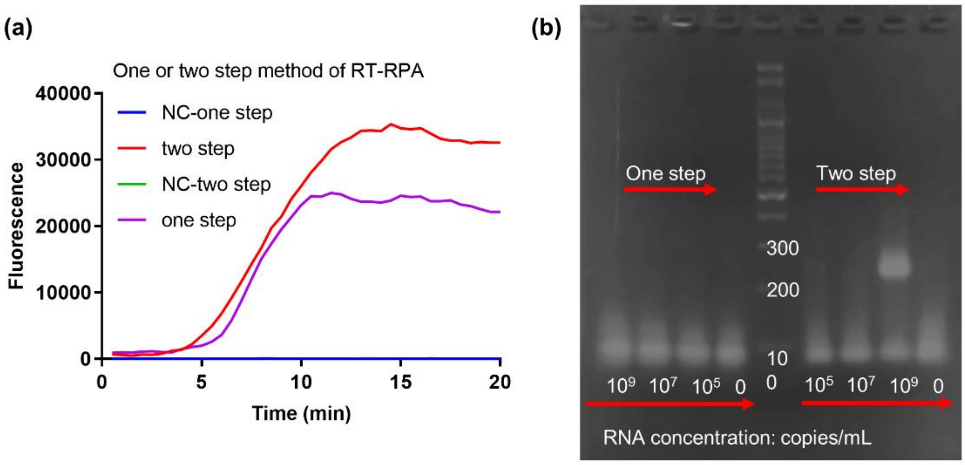

Figure S9. Comparison of the amplification efficiency bewteen one-step method and two-step method of RT-RPA. (a) Real-time detection of the exo RT–RPA reaction for two-step or one step through fluorescence instrument. (b) Agarose gel electrophoresis analysis of the exo RT–RPA reaction for two-step or one step.

Table S1. Primer and probe sequences of RPA reaction

**Name**	**Oligonucleotide sequence [5′-3′]**
ORF1ab-F	ACACCGGAAGCCAATATGGATCAAGAATCC
ORF1ab-R	ACCCACAGGGTCATTAGCACAAGTTGTAGG
UreB-F	CCTTCTGCAATCAATCATGCGTTAGATGTTGC
UreB-R	CCACCAGCGCCTTCAGTGTGGAAAGTGTGC
RNase P-F	AGCCACTGACTCGGATCCGCAACAACTCAGCC
RNase P-R	TGCGAAGAGCCATATCACGGAGGGGATAAGTG
ORF1ab-P	ATCGTGTTGTCTGTACTGCCGTTGCCACA/i6FAMdT/A/idSp/A/iBHQ1dT/CATCCAAATCCTAAAGG
UreB-P	CTGCCATAGTGTCTTCTACACAACCGGCT/i6FAMdT/C/idSp/T/iBHQ1dT/CAAAGTGTCTGTGT
RNase P-P	CACATCCGAGTCTTCAGGGTCACACCCAAG/i6FAMdT/A/idSp/T/iBHQ1dT/GAAAAGACACTCCTC

F: Forward primer; R: Reverse primer; P: Probe.

4. Construction of the Portable Reader

The isothermal incubation unit of the portable reader was mainly two heating pads, controlled by a temperature circuit, sitting at the bottom and on the top lid of the chamber. Eight magnets on and around the lid are used to secure the heating pads to be fully in contact with the chip. A thermal insulating pad surrounding the chamber is employed to maximally prevent the heat loss from chip edges. The temperature probe was attached to the chamber bottom. With these improvements (Figure S10), our data suggested that the temperature of the heating chamber has been steadily kept at 40 ± 1 ºC for RPA, even when the environmental temperature changed from 4 to 37 ºC.

We further validated the temperature stability of our system. We conducted repeated test of 4×10^3 copies/mL SARS-CoV-2 samples under different local temperatures. And the results showed that there was no significant difference in detection results at a local temperature of 10, 20 or 30 ℃ (Figure S12).

Table S2. The cost of the portable reader

Items	Cost $
Temperature controller	3
Heating pad	1
Lamp	10
Rechargeable batteries and circuits	8
Plastic cabinet	5
**Total**	**27**

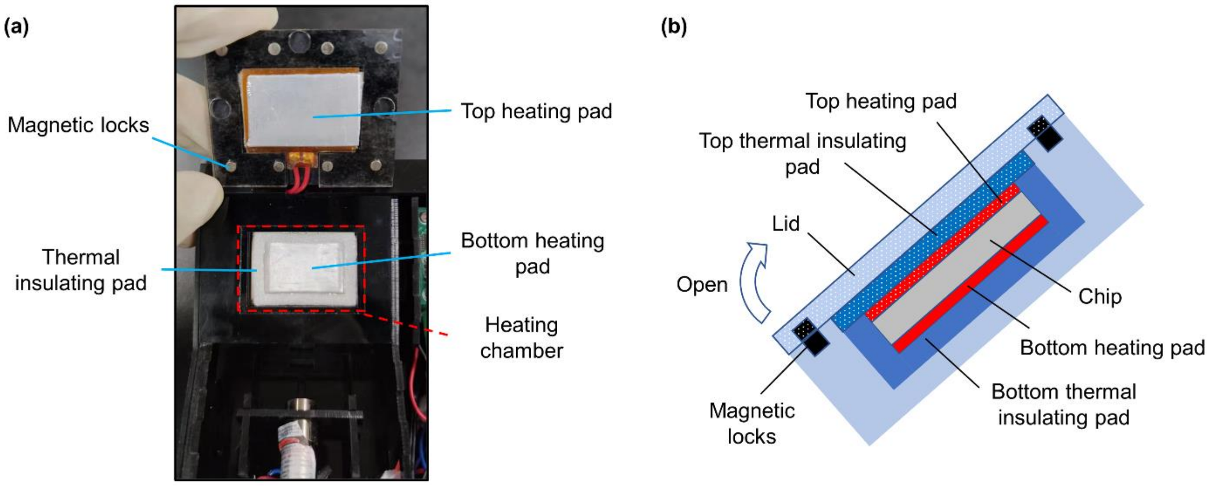

Figure S10. Structure illustration of the heating chamber (side view, closed state). (a) The side view of the heating chamber. (b) The closed state of the heating chamber.

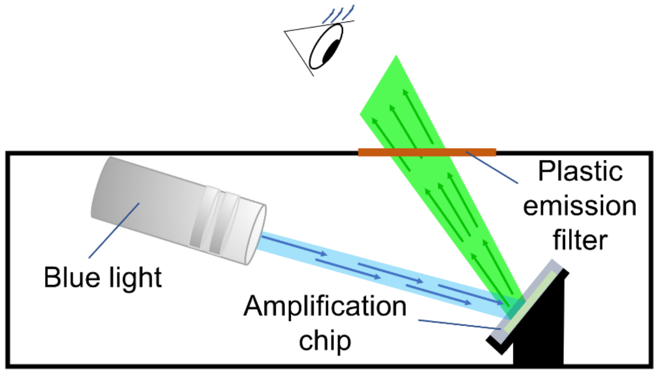

Figure S11. The visual readout unit of the portable reader. The plastic emission filter can pass the green fluorescence and blocking exciting light from blue light, to makes easier for users to visually differentiate green light signal from background light.

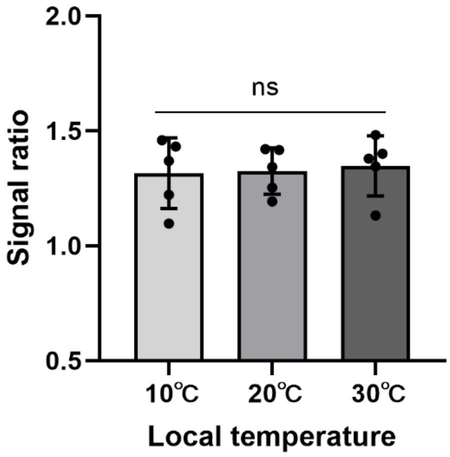

Figure S12. The temperature stability of our system. There was no significant difference in detection results at a local temperature of 10, 20 or 30 °C. Five replicates were run (n=5). The signal ratio represents the ratio of fluorescence values of positive and negative controls in a batch of tests. Error bars represent the means ± SD from replicates. (ns: not significant, t-test).

5. Construction of Amplification Chip

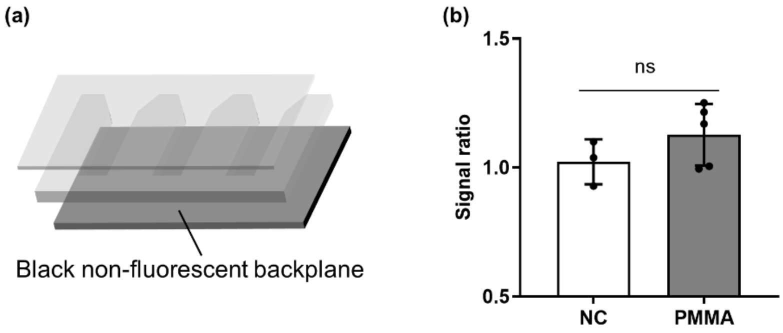

Figure S13. Design of the amplification chip and selection of inner material. (a) The amplification chip was made of three layers of PMMA plate, and the background negative plate was a black non-fluorescent plate to reduce light reflection and improve the signal-to-noise ratio. (b) Direct passivation of the PMMA surface is not good enough for the further RPA reaction. (ns: not significant).

6. Performance of the Platform Using Contrived Samples

We repeated the tests using contrived samples, and the analytical performance of this work meet or exceed the clinical testing requirements. For the detection of SAR-CoV-2, the LoD is as low as 400 copies/mL, which exceed the virus load (10^4 to 10^7 copies per mL) in clinical nasopharyngeal swabs samples [6-8]. For the detection of *H. pylori*, the LoD can reach to 10^3

counts/mL, comparable to the bacterial load (10^2 ~ 10^3 CFU/mL) in typical clinical saliva samples [9-10].

Table S3. Performance of the platform for SARS-CoV-2 contrived sample

SARS-CoV-2						LoD
Concentration (copies/mL)	4×10^1	4×10^2	4×10^3	4×10^4	4×10^5	4×10^2 copies/mL
Replicates	0/3	15/21	15/19	6/8	3/3	
Positive consistency rate	0	71.43 %	78.95 %	75 %	100 %	
Coefficient of variation (CV)	2.263 %	9.264 %	12.75 %	5.185 %	12.65 %	

Table S4. Performance of the platform for *H. pylori* contrived sample

H. pylori				LoD
Concentration (counts/mL)	10^2	10^3	10^4	10^3 counts/mL
Replicates	0/3	7/9	13/13	
Positive consistency rate	0	77.78 %	100 %	
Coefficient of variation (CV)	7.609 %	6.165 %	10.26 %	

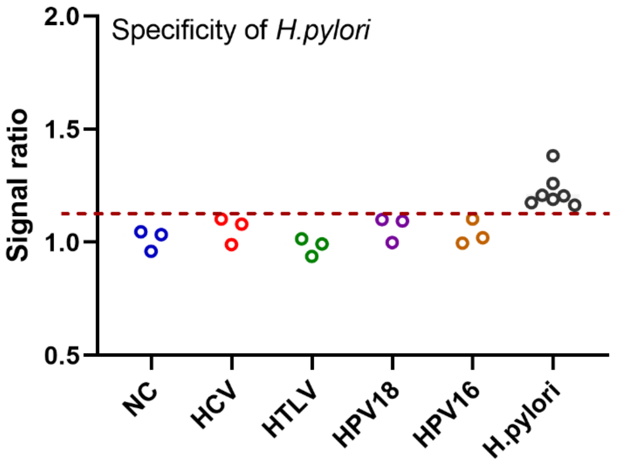

Figure S14. The specificity of our POCT platform for *H. pylori*. The plasmids of human papillomavirus (HPV) 16 and 18 types, hepatitis C virus (HCV), and human T-cell leukemia virus (HTLV) were used as templates to verify the specificity of this POCT platform for *H. pylori*. Each experiment was repeated at least three times (n≥3), and none generate fluorescent signal. The signal ratio represents the ratio of fluorescence values of positive and negative controls in a batch of tests. The value of average fluorescence intensity of negative control plus 3 × standard deviation (SD) which was used as the cut-off value for a positive signa.

7. Smart phone assisted reading and sharing of test results

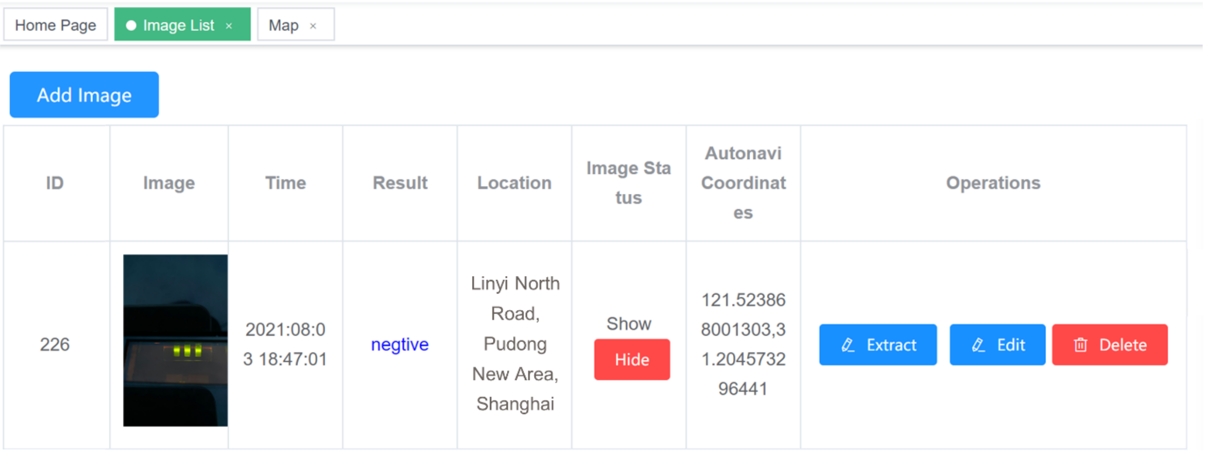

Figure S15. Simulation supportting website. Users can authorize and upload the test result image to the website cloud, and the website can extract the result information, GPS, time, etc., and directly feedback it to the medical and health institution, so that the user can receive treatment quickly, and also reduce the privacy leakage of the intermediate links. (website link: http://ditu1.vue100.com/ditu-admin/dist/#/kami/ditu1)

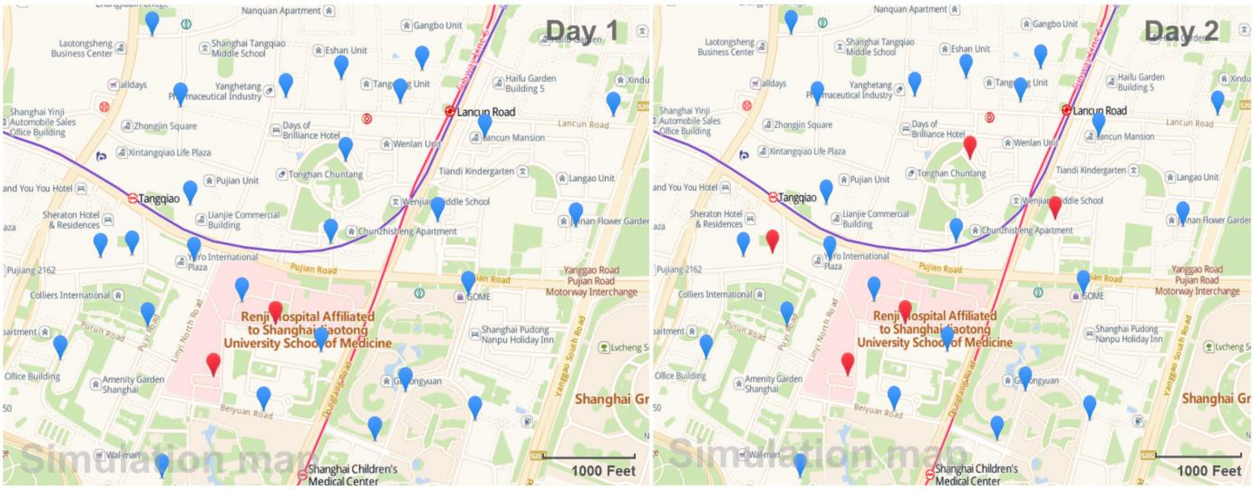

Figure S16. Simulation map of the spread of the pathogens in the community. The website can read the time and coordinates information of the result image and represent these on the map. Blue dots represent the negative, and red dots represent the positive. This map can accurately locate the relative position of images taken in adjacent residential buildings. We use factual GPS information and virtual detection results to display simulated maps.

8. Comparison with some typical point-of-care detection work

Table S5. Comparison with some typical point-of-care detection methods on SARS-CoV-2 with minimal instruments.

Item	Sample lysis	Equipment	Power-supply	One-pot	Sample-to-answer time	LoD of clinical/ contrived samples
This work	Room temperature	Portable reader ($27, battery life: 8-9hours)	Battery	Yes	30 min	400 copies/mL
CRISPR/Cas13a combines RT-RAA [11]	-	Intelligent miniature testing instrument (< $500), heater (< $50)	AC power	No	40 min (not include extraction)	-
ECS-CRISPR [12]	-	LED/UV light, or self-developed portable fluorescence detection device	-	Yes	25 min (not include extraction)	-
Paper microdevice [13]	Room temperature	Integrated paper microdevice, heat block and pipettes (deduced)	AC power	No	60 min	-
miSHERLOCK [14]	95℃	miSHERLOCK device ($15)	Battery	Yes	55 min	1000 copies/mL
RT-LAMP assay on POC system [15]	95 ℃	Heat block, handheld POC instrument	Battery	Yes	< 40 min	50000 copies/mL
STOPCovid.v2 [16]	95 ℃ / Room temperature	Heat block, two pipettes	AC power	Yes	< 60 min	33 copies/mL
AIOD-CRISPR [17]	Room temperature	LED transilluminator, pipette	AC power	Yes	< 60 min	-
SHINE [18]	Heating	Heat block, transilluminator, pipettes (deduced)	AC power	Yes	50 min	5000 copies/mL
eRPA [19]	95 ℃	Thermal mixer, two pipettes	AC power	No	45 min	-
CRISPR-based DETECTR [20]	-	Two heat blocks, pipettes	AC power	No	45 min	10000 copies/mL

- Not mentioned or explicitly defined

Table S6. Comparison with some isothermal nucleic acid amplification test devices.

Reference	Targets	Tests per batch	Heating	Batch on a charge	Time (min)	LoD
Lisa K. Lafleur, et al. 2016 [21]	DNA (MRSA)	1	95 and 50 °C, by battery	-	60	600 copies per action
Ruihua Tang, et al.2017 [22]	DNA (Salmonella)	1	65 °C by battery	Single use	60	10^2 ~ 10^3 CFU/ml
Elizabeth A. Phillips, et al. 2019 [23]	RNA (HIV-1)	1	65 °C by battery	Single use	90	3×10^5 copies/mL
Akkapol Suea-Ngam, et al. 2020 [24]	DNA (MRSA)	8	95 and 58 °C with hot-plate, by AC power	-	30	10^3 copies/mL
Carlos Manzanas, et al. 2021 [25]	RNA (SARS-CoV-2, influenza A)	8	62.5 °C, by battery-powered coffee mug	<3 batches (estimated)	50	< 10 genome equivalents per reaction
Dohwan Lee, et al. 2021 [26]	RNA (SARS-CoV-2)	1	68 °C by CaO powder	Single use	60	5×10^4 copies/mL
**This work**	**RNA (SARS-CoV-2) and DNA (*H. pylori*)**	**3**	**40 °C by battery**	**Up to 24 batches**	**30**	**4×10^2 copies/mL (SARS-CoV-2), 10^3 counts/mL (*H. pylori*)**

- Not mentioned or explicitly defined

[1] M. G. Mason, J. R. Botella, *Nature Protocols* **2020**.
[2] J. A. Cardona-Ospina, M. F. Villalba-Miranda, L. A. Palechor-Ocampo, L. I. Mancilla, J. C. Sepúlveda-Arias, *Prev Vet Med* **2019**, *172*, 104772.
[3] L. Mautner, C. K. Baillie, H. M. Herold, W. Volkwein, P. Guertler, U. Eberle, N. Ackermann, A. Sing, M. Pavlovic, O. Goerlich, U. Busch, L. Wassill, I. Huber, A. Baiker, *Virology Journal* **2020**, *17* (1), 160.
[4] J. M. Winchell, S. L. Mitchell, *Methods in molecular biology (Clifton, N.J.)* **2013**, *943*, 149.
[5] K. A. Curtis, D. Morrison, D. L. Rudolph, A. Shankar, L. S. P. Bloomfield, W. M. Switzer, S. M. Owen, *Journal of Virological Methods* **2018**, *255*, 91.
[6] R. Nouri, Z. Tang, M. Dong, T. Liu, A. Kshirsagar, W. Guan, *Biosens. Bioelectron.* **2021**, *178*, 113012.
[7] J. Fajnzylber, J. Regan, K. Coxen, H. Corry, C. Wong, A. Rosenthal, D. Worrall, F. Giguel, A. Piechocka-Trocha, C. Atyeo, S. Fischinger, A. Chan, K. T. Flaherty, K. Hall, M. Dougan, E. T. Ryan, E. Gillespie, R. Chishti, Y. Li, N. Jilg, D. Hanidziar, R. M. Baron, L. Baden, A. M. Tsibris, K. A. Armstrong, D. R. Kuritzkes, G. Alter, B. D. Walker, X. Yu, J. Z. Li, *Nat. Commun.* **2020**, *11* (1), 5493.
[8] X. Wang, L. Tan, X. Wang, W. Liu, Y. Lu, L. Cheng, Z. Sun, *Int J Infect Dis* **2020**, *94*, 107.
[9] J. K. C. Yee, *Exp. Mol. Med.* **2017**, *49* (11), e397.
[10] K. Bangpanwimon, P. Mittraparp-arthorn, K. Srinitiwarawong, N. Tansila, *J. Microbiol. Biotechnol.* **2021**, *31* (4), 501.
[11] Q. Zhang, J. Li, Y. Li, G. Tan, M. Sun, Y. Shan, Y. Zhang, X. Wang, K. Song, R. Shi, L. Huang, F. Liu, Y. Yi, X. Wu, *Biosens. Bioelectron.* **2022**, *202*, 113978.
[12] F. Hu, Y. Liu, S. Zhao, Z. Zhang, X. Li, N. Peng, Z. Jiang, *Biosens. Bioelectron.* **2022**, *202*, 113994.
[13] V. P. Dinh, N. Y. Lee, *Biosens. Bioelectron.* **2022**, *204*, 114080.
[14] H. de Puig, R. A. Lee, D. Najjar, X. Tan, L. R. Soekensen, N. M. Angenent-Mari, N. M. Donghia, N. E. Weckman, A. Ory, C. F. Ng, P. Q. Nguyen, A. S. Mao, T. C. Ferrante, G. Lansberry, H. Sallum, J. Niemi, J. J. Collins, *Sci Adv* **2021**, *7* (32), eabh2944.
[15] A. Ganguli, A. Mostafa, J. Berger, M. Y. Aydin, F. Sun, S. A. S. de Ramirez, E. Valera, B. T. Cunningham, W. P. King, R. Bashir, *Proc. Natl. Acad. Sci. U.S.A.* **2020**, *117* (37), 22727.
[16] J. Joung, A. Ladha, M. Saito, N.-G. Kim, A. E. Woolley, M. Segel, R. P. J. Barretto, A. Ranu, R. K. Macrae, G. Faure, E. I. Ioannidi, R. N. Krajeski, R. Bruneau, M.-L. W. Huang, X. G. Yu, J. Z. Li, B. D. Walker, D. T. Hung, A. L. Greninger, K. R. Jerome, J. S. Gootenberg, O. O. Abudayyeh, F. Zhang, *N. Engl. J. Med.* **2020**, *383* (15), 1492.
[17] X. Ding, K. Yin, Z. Li, R. V. Lalla, E. Ballesteros, M. M. Sfeir, C. Liu, *Nat. Commun.* **2020**, *11* (1), 4711.
[18] J. Arizti-Sanz, C. A. Freije, A. C. Stanton, B. A. Petros, C. K. Boehm, S. Siddiqui, B. M. Shaw, G. Adams, T. F. Kosoko-Thoroddsen, M. E. Kemball, J. N. Uwanibe, F. V. Ajogbasile, P. E. Eromon, R. Gross, L. Wronka, K. Caviness, L. E. Hensley, N. H. Bergman, B. L. MacInnis, C. T. Happi, J. E. Lemieux, P. C. Sabeti, C. Myhrvold, *Nat. Commun.* **2020**, *11* (1), 5921.
[19] J. Qian, S. A. Boswell, C. Chidley, Z. X. Lu, M. E. Pettit, B. L. Gaudio, J. M. Fajnzylber, R. T. Ingram, R. H. Ward, J. Z. Li, M. Springer, *Nat. Commun.* **2020**, *11* (1), 5920.
[20] J. P. Broughton, X. Deng, G. Yu, C. L. Fasching, V. Servellita, J. Singh, X. Miao, J. A. Streithorst, A. Granados, A. Sotomayor-Gonzalez, K. Zorn, A. Gopez, E. Hsu, W. Gu, S.

Miller, C.-Y. Pan, H. Guevara, D. A. Wadford, J. S. Chen, C. Y. Chiu, *Nat. Biotechnol.* **2020**, *38* (7), 870.
[21] L. K. Lafleur, J. D. Bishop, E. K. Heiniger, R. P. Gallagher, M. D. Wheeler, P. Kauffman, X. Zhang, E. C. Kline, J. R. Buser, S. Kumar, S. A. Byrnes, N. M. Vermeulen, N. K. Scarr, Y. Belousov, W. Mahoney, B. J. Toley, P. D. Ladd, B. R. Lutz, P. Yager, *Lab Chip* **2016**, *16* (19), 3777.
[22] R. Tang, H. Yang, Y. Gong, M. You, Z. Liu, J. R. Choi, T. Wen, Z. Qu, Q. Mei, F. Xu, *Lab Chip* **2017**, *17* (7), 1270.
[23] E. A. Phillips, T. J. Moehling, K. F. K. Ejendal, O. S. Hoilett, K. M. Byers, L. A. Basing, L. A. Jankowski, J. B. Bennett, L. K. Lin, L. A. Stanciu, J. C. Linnes, *Lab Chip* **2019**, *19* (20), 3375.
[24] A. Suea-Ngam, I. Choopara, S. Li, M. Schmelcher, N. Somboonna, P. D. Howes, A. J. deMello, *Adv Healthc Mater* **2021**, *10* (7), e2001755.
[25] C. Manzanas, M. M. Alam, J. C. Loeb, J. A. Lednicky, C. Y. Wu, Z. H. Fan, *ACS Sens* **2021**, *6* (11), 4176.
[26] D. Lee, C. H. Chu, A. F. Sarioglu, *ACS Sens* **2021**, *6* (9), 3204.
